# Leiomyosarcomas of Vascular Origin in the Extremity

**DOI:** 10.1155/2009/385164

**Published:** 2009-06-29

**Authors:** R. Abed, A. Abudu, R. J. Grimer, R. M. Tillman, S. R. Carter, L. Jeys

**Affiliations:** The Royal Orthopaedic Hospital, Bristol Road South, Birmingham B31 2AP, UK

## Abstract

Between 1996 and 2006 a total of 278 patients with soft tissue Leiomyosarcoma were treated at our centre. We identified 16 patients (5.8%) where the tumour directly arose from the blood vessels. These tumours were studied to determine their prognosis and behaviour. All tumors were in the lower limbs: 11 from the femoral vein, 3 popliteal vein, and 2 from the posterior tibial vein. Mean tumour size was 10.4 cm (3 to 33). Histological grade was high in all patients. Surgical treatment was amputation in one, excision with or without vascular reconstruction in 12 followed by radiotherapy, and 3 patients had no surgery because of advanced disease at diagnosis. Seven out of the 16 patients (44%) had metastasis at diagnosis, and five patients without metastasis at diagnosis rapidly developed metastases at a median time of 5 months from diagnosis (2–30 months). The overall survival of the patients at 5 years was 25% which was considerably worse than those with nonvascular leiomyosarcoma. We conclude that patients with leiomyosarcoma of vascular origin have a very high risk of metastases and poor prognosis when treated in the conventional way.

## 1. Introduction

 Leiomyosarcomas arising directly from the blood vessels are rare. Hence, there is limited publication and experience of the clinical presentation and results of treating this type of tumour. The tumours arise directly from the muscular wall of either a major vein or artery. The majority of the tumours arising in the extremities affect the femoral vascular bundle [1]. In general, leiomyosarcomas can be divided into three types according to their site of origin [1]. Leiomyosarcomas of soft tissues, being the most common [2], cutaneous leiomyosarcoma having the best prognosis [3], and vascular leiomyosarcomas. 

Vascular leiomyosarcomas are defined as any leiomyosarcoma that arise from a major vessel based on clinical picture, radiographic findings, and pathological findings [2]. There are only a few reports of leiomyosarcomas of vascular origin with the majority being isolated case reports [3–8]. There is also controversy about the prognosis of patients with this type of lieomyosarcoma [1, 9, 10]. The majority of patients are at present treated like most soft tissue sarcomas with limb preserving surgery and radiotherapy. 

The aim of our study is to report our experience of the clinical presentation, results of surgical treatment, and oncological outcome of patients with vascular leiomyosarcoma treated at a single supraregional oncology centre to determine whether the current treatment protocol for this tumour is adequate or not. 

## 2. Materials and Methods

Between 1996 and 2006, 278 patients with leiomyosarcoma were treated at our centre which is a supraregional centre for the treatment of bone and soft tissue sarcomas. We identified 16 patients with leiomyosarcoma of vascular origin from the prospectively collected orthopedic oncology database. The minimum follow-up was 12 months. The following information was collected retrospectively from the case records of the oncology database: the gender, age, presentation, duration of complaint, grade of tumor, stage of disease, type of surgical procedure, surgical margins, surgical complications, whether they had vascular reconstruction, any adjuvant treatment such as radiotherapy or chemotherapy, follow up duration, subsequent recurrence or metastasis, and condition of the patient at present.

The tumour grade was according to the FNCLCC system proposed by Trojani et al. [11] and the stage and margins of resection were according to the Enneking staging system [12]. Survival analysis was performed using Kaplan-Meier's method. 

## 3. Results

### 3.1. Clinical Presentation

**Figure 1:**
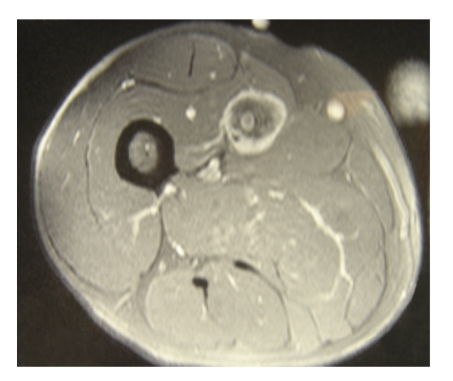
MRI picture of Leiomyosarcoma arising from the femoral vein.

**Figure 2:**
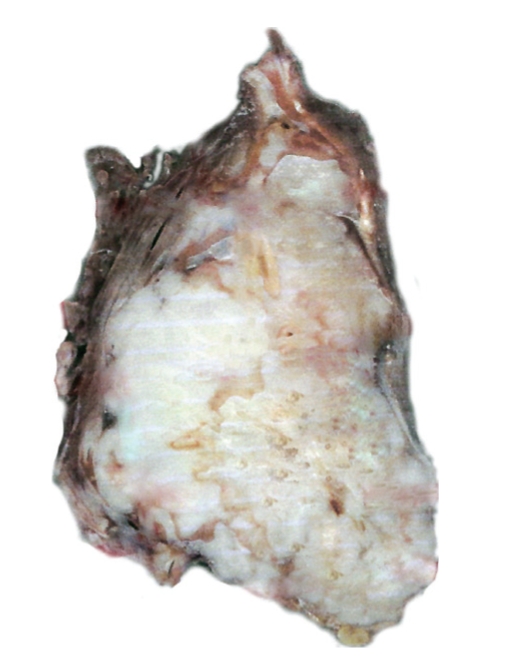
Macroscopic picture of leiomyosarcoma of vascular origin.

Of the 278 patients with leiomyosarcoma of the extremities treated at our centre over the study period, only 16 patients accounting for 5.8% of the total were classified as vascular leiomyosarcomas. There were 9 females and 7 males. The average age was 67 years (range 51–77). The tumours were located in the lower extremity in all the patients arising from the femoral vein in 11 patients (Figures 1  and 2), popliteal vein in three, and posterior tibial vein in 2 patients. 

Delay in diagnosis was common with eight patients (50%) presenting with significant leg swelling and edema without any palpable soft tissue lump and clinically diagnosed as suffering from deep venous thrombosis. The tumours were diagnosed in these patients during ultrasound and/or venography performed to investigate them for deep venous thrombosis. Six patients presented with lumps in the groin, thigh, popliteal fossa, and ankle, two of these underwent unplanned excision (“whoops procedure”) at another institution prior to referral to our centre. One patient presented with hemoptysis from lung metastasis, and the tumour was only diagnosed during staging investigations. One patient presented with hematoma in the thigh. Metastatic disease was evident in 7 patients (44%) at the time of diagnosis. The clinical presentation of the patients is summarized in Table 1. 

**Figure 3:**
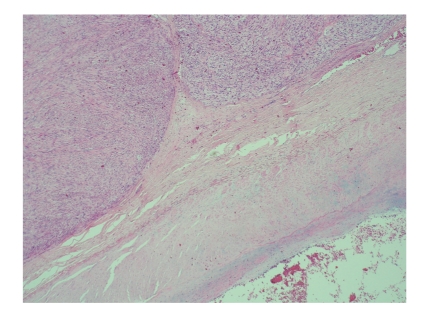
Miscroscopic picture of leiomyosarcoma arising from major blood vessel.

All tumours were high grade (Figure 3). The clinical stage of the disease at diagnosis was stage IIB in nine patients (56%) and stage IIIB in 7 patients (44%). The tumor size varied from 3 to 33 cm (median 9 cm). 

### 3.2. Treatment

The treatment plan for each patient was determined by a multidisciplinary team. Three patients with advanced disease at diagnosis were treated with palliative intent while 13 patients were treated with curative intent with surgery involving resection and/or without vascular reconstruction in these patients. Surgical treatment was amputation in one and limb preserving in 12 patients. 

Excision margins were classified as wide in 3 patients, marginal in 7, and intralesional in 3 patients according to the Musculoskeletal society classification system [12]. There was no failure of vascular reconstruction. One patient had postoperative wound infection and necrosis which required further surgery with wound debridement and irrigation.

Of the 12 patients with limb preservation, 7 required excision of the main artery en bloc with the tumour and vein in order to get clearance. Arterial reconstruction using a vein interpositional graft was performed in these patients. None of the patients had vein reconstruction as we often tried to preserve the saphenous veins in these patients with the hope that these would become the dominant venous drainage. This was determined preoperatively, and the surgery was done in collaboration with a vascular surgeon who performed the reconstruction. In the remaining 5 cases the resection included only the vein and no reconstruction was done. This was possible in case of resection of the femoral vein due to the presence of the saphenous vein which together with the collaterals compensated for the loss of the femoral vein.

All patients who had resection had postoperative radiotherapy, and two of the patients who did not have surgery had palliative radiotherapy. Two patients had postoperative chemotherapy because of metastases at diagnosis. 

### 3.3. Oncological Results

At the time of review, one patient had died of lung carcinoma unrelated to the vascular leiomyosarcoma. Three patients were alive and free of disease at a mean time of 27 months (range 21–36), one was alive with metastases and 11 had died of the disease at a mean time 14 months (range 2–36). The cumulative overall survival was 25% at 5 years. Of the patients with localized disease at diagnosis, the cumulative metastases free disease was 33% and overall survival was 44% at 5 years. One patient developed recurrence 5 months after hip disarticulation. The amputation was done for progressive thigh hematoma. This patient was diagnosed with stage 3B at diagnosis and received postoperative palliative chemotherapy, and the patient died 9 months after diagnosis. 

## 4. Discussion

In general, Leiomyosarcomas are uncommon tumors and thought to have poor long-term prognosis. Svarvar et al. reported on 225 patients with leiomyosarcoma of all types from the Scandinavian Sarcoma Group with a cumulative survival of 49% at 10 years [13]. 

Our study has focused specifically on Leiomyosarcomas of vascular origin. Our experience confirms that these tumours are very rare, accounting for only 5.8% of all patients with leiomyosarcoma treated at our centre. It is therefore not surprising that experience in treating this type of tumour is limited, and most reports in literature are isolated case reports [3–8].

The largest series to date is from Kevorkian and Cento who reported on 86 patients with leiomyosarcoma arising from a major blood vessel in the 1970s [14]. The majority of the tumours in their series arose in large-to-medium-sized vessels 35 tumours (41%), 33 tumours (38%) arose in the inferior vena cava, 10 tumours (12%) in the pulmonary artery, and 8 tumours (9%) in the systemic arteries. Dzsinich et al. [15] reported on 13 cases of primary venous leiomyosarcoma. 8 of the 13 cases arose from the inferior vena cava, 2 from the iliac vein, 1 from the ovarian vein, and 2 from the great saphenous vein.

The above series are different from the patients in our series in that we have only studied patients with extremity vascular leiomyosarcomas. The outcome reported in our series is very similar to those reported by Berlin et al. [1] who studied 6 patients with leiomyosarcoma of venous origin in the extremities. The tumours in Berlin's series were located in the superficial femoral vein in 3 cases, in the great saphenous vein in one case, in the popliteal vein in one case, and in the axillary vein in one case. Five patients in that series died of metastatic disease, and one was still alive with metastatic disease within 5 years of treatment.

The behaviour of vascular leiomyosarcomas has been reported by other authors. Leu and Makek [9] reported good prognosis on 5 cases of intramural venous leiomyosarcomas, and Hadju [10] reported low metastatic potential. On the other hand all the cases reported by Berlin et al. [1] had metastases with 5 cases dying from metastatic disease within 5 years. The results of our series showed a very poor outcome in vascular leiomyosarcoma with 75% of patients died of metastatic disease within the first 3 years of diagnosis, although good local control by surgery and radiotherapy was achieved. This is very similar to the series of Berlin et al. [1].

Our experience is that about half of the patients with leiomyosarcomas of vascular origin had metastatic disease at diagnosis which indicated either a very aggressive course of the disease or the effects of delay in diagnosis as most patients had been thought to have deep venous thrombosis resulting in diagnostic delay. Of the 9 patients without metastases at diagnosis 5 patients developed metastases within 36 months of the disease indicating that the tumours have an aggressive clinical course. The cumulative survival at 5 years was rather poor at 25%. It is unclear whether chemotherapy would have made a difference in the prognosis of these patients. Only two patients had chemotherapy and that was palliative for metastatic disease.

Good local control can often be achieved with surgical excision of the tumour with en-bloc resection of the vessels combined with radiotherapy. Our experience also leads us to believe that reconstruction of the vein is not necessary in those cases where a major vein such as the long saphenous vein in the lower limb can be preserved. This together with collateral venous circulation is often enough for venous drainage. Other authors have reported a high thrombosis rate in those who undergo venous reconstruction [16].

We conclude that patients with leiomyosarcoma arising from the blood vessels have a high risk of presenting with metastases at the time of diagnosis or early after diagnosis when treated in the conventional way used to treat other soft tissue sarcomas. It is unclear whether a more aggressive treatment regime will improve the prognosis of these patients, and we believe that this can best be answered by a multiinstitutional study using novel treatment strategy.

## Figures and Tables

**Table 1:** The clinical presentation in patients with leiomyosarcoma of vascular origin. NA = Not available.

Gender	Age	Presentation	Location	Size (cm)	Grade	Stage
F	55	Leg swelling	Thigh	NA	3	2B
F	74	Leg swelling	Thigh	13	3	2B
M	67	Lump in ankle	Ankle	3.5	2	2B
F	51	Hematoma in the thigh	Thigh	33	3	3B
F	54	Lump in popliteal fossa	Popliteal fossa	3	2	2B
M	60	Leg swelling	Thigh	9	3	2B
M	67	Lump in groin	Thigh	NA	3	2B
M	68	Leg swelling	Calf	NA	2	2B
M	77	Leg swelling	Popliteal fossa	NA	3	3B
F	63	Thigh swelling	Thigh	NA	3	3B
F	63	Lump in popliteal fossa	Popliteal fossa	13	3	3B
M	75	Lump in thigh	Thigh	9	3	2B
M	72	Haemoptysis	Thigh	9	3	3B
F	72	Lump in thigh	Thigh	9.5	3	3B
F	68	Leg swelling	Thigh	9	3	2B
F	75	Leg swelling	Thigh	9	3	3B
